# Does Seasonal Variation in Agility of Avian Prey Influence Body Size of Breeding Male Cooper's Hawks? And Comments on the Putative Influence of Avivory on Reproductive Output in Cities

**DOI:** 10.1002/ece3.72837

**Published:** 2026-01-09

**Authors:** Robert N. Rosenfield, Andrew C. Stewart, Paul N. Frater, Eric L. Holmgren

**Affiliations:** ^1^ Department of Biology University of Wisconsin‐Stevens Point Stevens Point Wisconsin USA; ^2^ Retired Cobble Hill British Columbia Canada; ^3^ Wisconsin Department of Natural Resources Wisconsin Rapids Wisconsin USA; ^4^ Retired Deephaven Minnesota USA

**Keywords:** *Astur cooperii*, body size, climate change, natural history, prey agility, reproductive output, tracking prey size, urbanization

## Abstract

Recent studies have modeled predator–prey and niche dynamics in the avivorous, forest adapted Cooper's Hawk (*Astur cooperii*) to explain its recent and rapid colonization, including high reproductive output in varied, especially urban environments across North America. We contend that such research is compromised in part because of untenable assumptions and understudied aspects of inter‐population variation in the diet and foraging ecology of breeding males who predominately catch food for their young and markedly larger mates. These inadequacies are aggravated by unfounded generalizations in the literature of this species' diet, including a notable lack of accounting for the plausible link between inter‐population variation in body mass (size) of breeding males and the size and hence agility of their avian prey. Ecologists have dismissed the hypothesis that a males' smaller size and hence its increased agility enhances foraging efficiency because during the nestling stage males typically catch inexperienced, easy‐to‐catch nestlings and fledglings, prey for which agility seems unnecessary. However, our findings support this hypothesis because during the crucial and rarely studied pre‐incubation stage British Columbia and Wisconsin males predominately caught experienced adult birds. Our pre‐laying diet data also support the principle that predators match the size of their prey because smaller British Columbia hawks predominately caught smaller, more agile birds vs. those taken by larger Wisconsin counterparts. We found similar and high proportions of avian prey and no statistical difference between similar and high production of nestlings between Cooper's Hawk nests in urban and rural environments in British Columbia, and therefore no support for the little researched and oft‐suggested premise of greater productivity in urban vs. rural environments due to supposed high abundance of suitable avian prey in cities. Our findings combined with comments regarding pertinent literature clarify concepts and expand our knowledge of the diet and foraging ecology of breeding Cooper's Hawks.

## Graphical abstract

Our findings regarding prey of breeding Cooper's Hawks in British Columbia and Wisconsin expand knowledge of their food habits and support the premise that seasonal agility of prey influences body size of nesting males. We deem it untenable to assume greater production of young at urban vs. rural nests based on proportion of avian prey use.

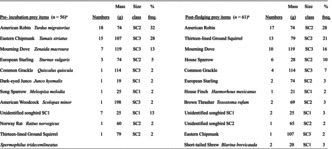

## 1 Introduction

Recent research on the predator–prey dynamics of Cooper's Hawk (*Astur cooperii*) is likely related to rapid increases in urban nesting populations of this avivorous, forest‐adapted species since about 1970 following its recovery from pesticide contamination and persecution from shooting (Cava et al. 2012; Stout and Rosenfield 2010; Rosenfield, Madden, et al. 2020; Rosenfield et al. 2022). The Cooper's Hawk is likely the most common backyard breeding, high‐trophic predator in cities across its broad, coast‐to‐coast North America range into southern Canada and central Mexico (Rosenfield et al. 2022). This crow‐sized raptor exhibits high reproductive indices and nesting densities in cities, putatively in part because urban environments may have high numbers of their predominant ground‐foraging avian prey, including small‐to‐medium‐sized songbirds (e.g., House Sparrrow [ *Passer domesticus* ], American Robin [*Turdus migratorious*]) and doves (e.g., Mourning Dove [ *Zenaida macroura* ]) (Rosenfield, Madden, et al. 2020; Morozov 2022); secondarily, small mammals, particularly ground‐dwelling squirrels (*Spermophilus* spp.) and chipmunks (e.g., *Tamias and Eutamias* spp.), are also common prey (Rosenfield, Madden, et al. 2020). The diet of nesting Cooper's Hawks is strongly tied to the foraging ecology of breeding males who establish nesting territories, hunt live prey and almost exclusively provision food for themselves, their markedly (~1.7×) larger mates, and young throughout the entire nesting cycle from pre‐incubation thru post‐fledging stages (Rosenfield, Madden, et al. 2020).

A breeding male's foraging success during the pre‐incubation period is especially key because it is the means to both procure a mate among prospecting females and to provide ample resources for egg formation, which sets the pair's annual reproductive potential (Millsap et al. 2013; Rosenfield, Madden, et al. 2020). We highlight that clutch size is the most important factor in determining reproductive success (and evolutionary potential) as it establishes an upper limit for the number of offspring that can be produced in any single bout of reproduction (Williams 1966; Thomson et al. 1998). Further, egg production is the most immediate productivity response to the seasonal arrival of avian prey for Cooper's Hawks (Rosenfield and Anderson 2016). Thus, following Snyder and Wiley (1976) we expect strong selective pressure on the foraging success of males during the crucial pre‐incubation stage, which period markedly lacks published dietary data (Rosenfield, Madden, et al. 2020).

Our multi‐decadal and geographically broad research of four northern breeding populations of Cooper's Hawks (from Wisconsin in central United States, west to coastal British Columbia, Canada) during overlapping years has established the existence of population‐specific body sizes, other anatomical attributes, and prey use which are likely due in part to adaptations to local environments (Rosenfield et al. 2010, 2024; Rosenfield, Stewart, et al. 2020). Indeed, we reported that a statistically significant east–west longitudinal clinal decrease in body mass (a reliable index to overall body size) of breeding male Cooper's Hawks among populations was unrelated to genetic drift or climate, but plausibly to the enhanced aerial agility, or flight performance (via acceleration and maneuverability) in smaller west‐central and even smaller western conspecifics who predominantly hunt birds, especially prey of smaller size which likely exhibit greater aerial agility than larger avian prey (Andersson and Norberg 1981; Rosenfield et al. 2010; Rosenfield, Stewart, et al. 2020; Sonsthagen et al. 2012). Notably, this east–west cline in male body size is concordant with decreasing size of avian prey delivered to nestling Cooper's Hawks among all four sites (Peterson and Murphy 1992; Rosenfield et al. 2010; Cava et al. 2012).

**FIGURE 1:**
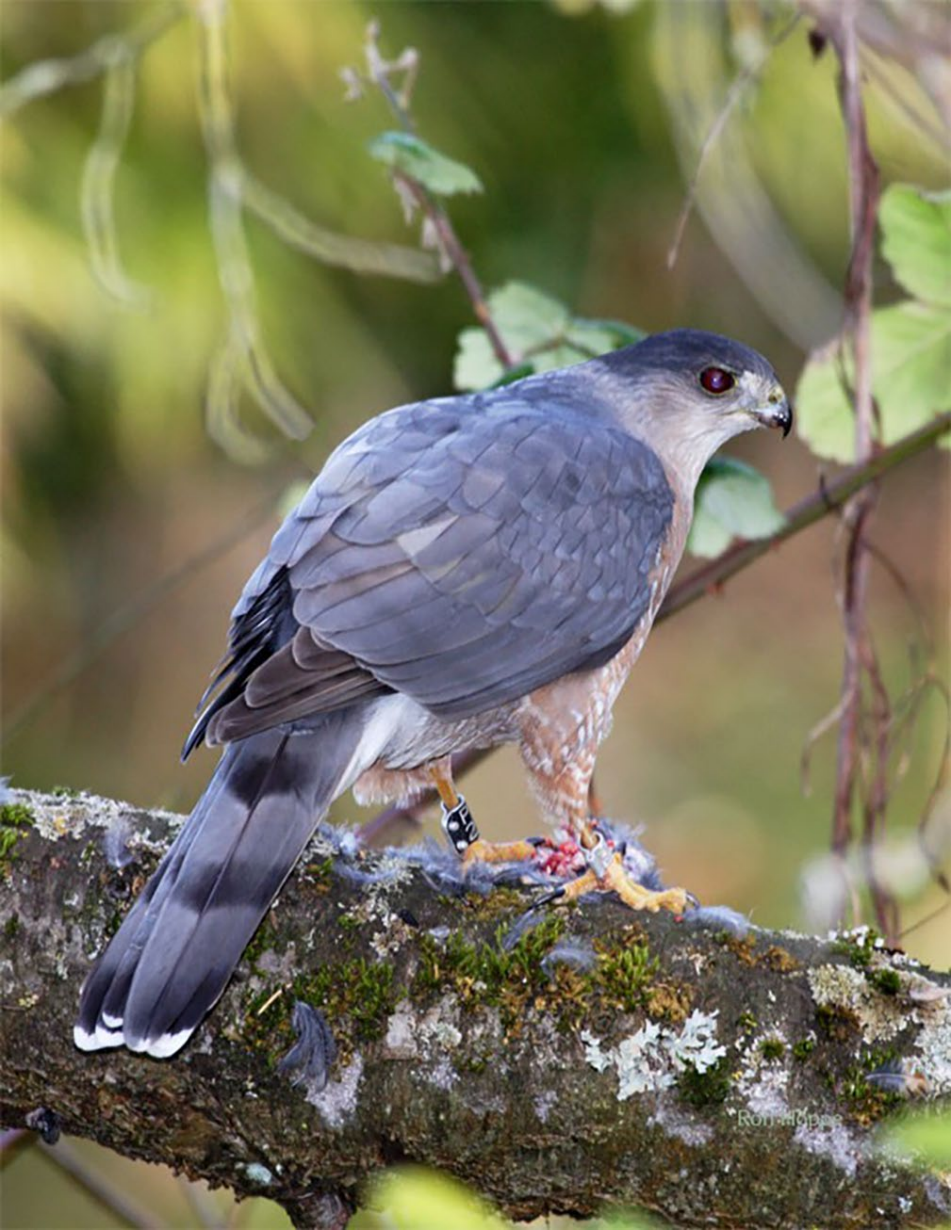
Adult male Cooper's Hawk with avian prey near its nest in Victoria, British Columbia. Photo by Ron Hoppe.

We highlight that predator–prey interactions in avivorous hawks are inherently body size‐dependent such that foraging success of predators via aerial agility depends on their ability to phenotypically match the size of their primary adult prey. This follows from the fact that agility increases with decreasing mass (size) such that, for example, hunting small, adult avian prey selects for enhanced aerial agility via smaller predator size (Wattel 1973; Pennycuick 1975; Andersson and Norberg 1981; Abrams 2000; Tornberg et al. 2014). Further, because of the agility of adult avian prey species, body size, wings, legs, toes, and talons of Cooper's Hawks are likely subject to strict canalizing influences (Figure 1; Whaley and White 1994; Brose 2010) including “severe” selective pressures on body size in males who prey on agile, relatively small songbirds in the Order Passeriformes (Snyder and Wiley 1976; Tornberg et al. 2014). Notably, the Cooper's Hawk exhibits one of the highest degrees of reversed‐size dimorphism among raptors (Rosenfield, Madden, et al. 2020).

Curiously, researchers have doubted and/or dismissed the premise that agility of breeding male Cooper's Hawks is important to prey selection because during the hawk's nestling stage, wherein most data exist for nesting diet studies of this species, breeding males in several studies mostly delivered inexperienced, temporally available, nestling and/or fledgling passerines of assumed low agility due to their relative immobility (nestlings) or underdeveloped evasive, anti‐predator flight behaviors (recent fledglings). These age cohorts represent avian prey for which predator agility may not always be necessary (Bielefeldt et al. 1992; Cava et al. 2012; Millsap et al. 2013; Schoenjahn et al. 2020). Thus, adult males might predominantly hunt easier to catch less agile bird species of larger size or, where available, less agile mammalian prey during the pre‐incubation stage.

That said, if during pre‐incubation males capture adult birds of the same species as young‐of‐the‐year taken during the hawk's nestling stage, then males would be seasonally obliged to track the escape tactics including greater agility in evasive maneuvering by experienced adult prey of similar size to younger counterparts. Such maneuvering would be adaptive in male Cooper's Hawks via the selective pressure of agility in small, adult avian prey on *Astur* morphology as proffered and mathematically demonstrated by Andersson and Norberg (1981). Further, these age‐related, seasonal predator–prey dynamics would strengthen our plausible explanation of a clinal decline in male body size being concordant with (and influenced by) a longitudinal increase in agility of smaller avian prey caught by breeding male Cooper's Hawks from Wisconsin to British Columbia (Rosenfield et al. 2010).

However, there is a marked lack of detailed studies of predator behavior and diet of Cooper's Hawks throughout their range (Cooper, Yeh, and Blumstein 2022; Rosenfield et al. 2022). And several diet investigations did not report and/or systematically quantify ages of prey, avian or otherwise, across the entire nesting cycle (e.g., Hamerstrom and Hamerstrom 1951; Craighead and Craighead 1956; Meng 1959; Kennedy and Johnson 1986; Estes and Mannan 2003; but see Cava et al. 2012). This is unfortunate because foraging tactics of breeding raptors are influenced by their seasonal use of young‐of‐the‐year prey species (e.g., Meese and Fuller 1989; Rosenfield et al. 1995). Additionally, and despite this lack of knowledge of prey use across various nesting stages and environments, researchers have oft suggested that there is greater productivity in urban vs. rural Cooper's Hawk nests due to supposed high abundance levels of suitable avian prey in cities (e.g., Kettel et al. 2019; Rullman and Marzluff 2014; Morozov 2022). However, there are few paired habitat studies documenting these suppositions (Kettel et al. 2018; Morozov 2022).

Herein we document: (1) size and age of prey species delivered by males during pre‐incubation in British Columbia and Wisconsin, and in the post‐fledging stage in Wisconsin, (2) species, age, and size of prey in rural British Columbia during incubation through post‐fledgling stages, and (3) productivity at urban and rural nests in British Columbia. Notably, our data do not support the premises that aerial agility is unimportant to male breeding Cooper's Hawks or that productivity is greater in urban vs. rural nests. We believe that our findings, combined with our comments regarding a review of pertinent literature, clarify underlying concepts, correct mischaracterizations, and expand our knowledge of the diet and foraging ecology of male breeding Cooper's Hawks.

## 2 Methods

### 2.1 Study Areas and Populations

Our study areas in Wisconsin, United States, and British Columbia, Canada, spanned ~2700 km across the northern part of the Cooper's Hawk's breeding range (43^o^–48^o^ N; Rosenfield et al. 2010).

### 2.2 Wisconsin

We studied prey use by breeding Cooper's Hawks in the ~42,408‐km^2^ southeastern quarter of Wisconsin, 1991–2023. Principal research sites therein included rural areas in Portage County including the adjacent municipalities of Stevens Point, Whiting, and Plover (44° N; 89° W), with a predominately urban population of ~38,000; and nests in the city of Oshkosh in Winnebago County (44° N; 88° W), with a human population of ~66,100. Our other primary field area included rural areas in and around the Kettle Moraine State Forest, South Unit in Waukesha and Jefferson Counties (42° N; 88° W). We define urban nests as those occurring within the municipal limits of cities; rural nests were those outside municipal city boundaries, typically in rural forested tracts ≥ 16 ha, usually with < 3 houses within 0.4 km of a nest. A breeding territory (hereafter territory) was defined as an area centered at nest sites of 800 m in diameter occupied by a breeding adult in one or more years (Rosenfield and Bielefeldt 1999). All study areas were chosen without preconceptions about the suitability of breeding habitat for Cooper's Hawks, including the availability of prey (Rosenfield et al. 2010; Rosenfield 2018). Wisconsin has a highly seasonal, mid‐continental climate. Further details including depictions of the distribution of our Wisconsin study areas and nests can be found in Rosenfield and Anderson (2016) and Rosenfield et al. (2019).

### 2.3 British Columbia

The 89‐km^2^ British Columbia (BC) study area in southern Vancouver Island, British Columbia, Canada (48° N; 123° W) included urban nests in the city of Victoria, which has an urban human population of ~240,000 people. Rural nests occurred in the Saanich Peninsula adjacent to and within 22 km north of our Victoria study area. As in Wisconsin, breeding territories were found without preconceptions of suitability of nesting habitat; territories and urban and rural nests are defined as above. Vancouver Island has a temperate coastal climate. Further descriptions of our insular study area can be found in Rosenfield et al. (2010) and Cava et al. (2012).

### 2.4 Field Techniques

Each year we find most nests (> 90%) in Wisconsin and British Columbia before incubation by listening for dawn vocalizations and using binoculars and spotting scopes to observe courting behaviors including nest construction by breeding pairs in March and April (Stewart et al. 1996; Rosenfield 2018). During our multi‐decadal research in British Columbia and Wisconsin, no breeding pairs begin incubation in March and with only two exceptions in Wisconsin in the first 2 weeks of April. About a third of our early breeding females typically start the 34‐day incubation in the last 10 days of April, and almost all females have begun incubation by about the first 10 days of May in both our study areas (RNR and ACS, pers. obs; we note that incubation typically starts before all eggs are laid, [Rosenfield et al. 2016; Rosenfield, Madden, et al. 2020]). Herein we define the pre‐incubation period as March through April; the incubation, nestling, and post‐fledgling stages occur in May, June, and July through early August, respectively (Rosenfield 2018; Rosenfield et al. 2024).

We report opportunistically observed whole or partially eaten prey delivered by males to females during the pre‐incubation stage, and to post‐fledging young in Wisconsin (Table 1). Prey items documented during pre‐incubation in Victoria, British Columbia were obtained via prey remains near nests and through direct observations of prey deliveries by males as re‐summarized here from Cava et al. (2012). Rural prey items in British Columbia were from incubation through post‐fledging stages (Table 2).

**TABLE 1:** Prey delivered by 56 uniquely marked breeding male Cooper's Hawks to females during pre‐incubation in southeast Wisconsin (one delivery in each of 56 territories; 35 urban, 21 rural), 1991–2023; and prey deliveries by another marked male during the post‐fledging stage in an urban, central Wisconsin territory, 2022. Total sample sizes of prey items are indicated by n; mass is estimated average. Excluding Mourning Dove, American Woodcock, and mammals, all prey items were songbirds (i.e., Order Passeriformes). Size class prey categories: SC1 ≤ 27 g, SC2 28–91 g, SC3 ≥ 92 g.

Pre‐ incubation prey items (*n* = 56)^a^	Numbers	Mass (g)	Size class	% freq.	Post‐fledging prey items (*n* = 61)^a^	Numbers	Mass (g)	Size class	% freq.
American Robin *Turdus migratorius*	18	74	SC2	32	American Robin	17	74	SC2	28
Eastern Chipmunk *Tamais striatus*	15	107	SC3	28	Thirteen‐lined Ground Squirrel	13	79	SC2	21
Mourning Dove *Zenaida macroura*	7	119	SC3	13	Mourning Dove	10	119	SC3	16
European Starling *Sturnus vulgaris*	3	74	SC2	5	House Sparrow	6	28	SC2	10
Common Grackle *Quiscalus quiscula*	1	114	SC3	2	Common Grackle	4	114	SC3	7
Dark‐eyed Junco *Junco hyemalis*	1	19	SC1	2	European Starling	2	74	SC2	3
Song Sparrow *Melospiza melodia*	1	25	SC1	2	House Finch *Haemorhous mexicanus*	1	21	SC1	2
American Woodcock *Scolopax minor*	1	198	SC3	2	Brown Thrasher *Toxostoma rufum*	2	69	SC2	3
Unidentified songbird SC1	7	25	SC1	13	Unidentified songbird SC1	2	25	SC1	3
Norway Rat *Rattus norvegicus*	1	60	SC2	2	Unidentified songbird SC2	1	65	SC2	2
Thirteen‐lined Ground Squirrel	1	79	SC2	2	Eastern Chipmunk	1	107	SC3	2
*Spermophilus tridecemlineatus*					Short‐tailed Shrew *Blarina brevicauda*	2	20	SC1	3

^a^ All pre‐incubation prey items were adults (total urban prey included 12 American Robins, 9 Eastern Chipmunks, 4 Mourning Doves, 2 European Starlings, 6 unidentified SC1 birds, 1 Common Grackle and 1 Dark‐eyed Junco). At least 29 (49%) post‐fledging prey items were young‐of‐the‐year, including 10 American Robins (8 fledglings, 2 nestlings), 1 House Finch (nestling), 2 Brown Thrashers (fledglings), 3 Common Grackles (fledglings), 1 European Starling (fledgling), 11 Thirteen‐lined Ground Squirrels, and 1 Eastern Chipmunk.

**TABLE 2:** Prey of rural breeding Cooper's Hawks during the incubation through post‐fledging stages in southeast Vancouver Island, British Columbia, 1995–2011. Total sample size of prey items via prey remains and direct observation is indicated by *n*; direct observation subsamples for each prey item occur in brackets; mass is estimated average. Excluding Green‐winged Teal, Violet‐green Swallow, California Quail, Rock Pigeon, Northern Flicker, and mammals, all species are songbirds (i.e., Order Passeriformes). Size class prey categories: SC1 ≤ 27 g, SC2 28–91 g, SC3 ≥ 92 g.

Prey items (*n* = 210)^a^	Numbers	Mass (g)	Size class	% frequency
American Robin *Turdus migratorius*	75 [10]	70	SC2	36
European Starling *Sturnus vulgaris*	35 [1]	74	SC2	17
House Sparrow *Passer domesticus*	14 [5]	28	SC2	7
House Finch *Haemorhous mexicanus*	7 [1]	21	SC1	3
Spotted Towhee *Pipilo maculatus*	6	41	SC2	3
Varied Thrush *Ixoreus naevius*	4	79	SC2	2
Bewick's Wren *Thryomanes bewickii*	3	10	SC1	1
Steller's Jay *Cyanocitta stelleri*	1 [1]	128	SC3	< 1
American Crow *Corvus brachyrhynchos*	1	387	SC3	< 1
Red‐winged Blackbird *Agelaius phoeniceus*	1	64	SC2	< 1
Swainson's Thrush *Catharus ustulatus*	1	30	SC2	< 1
Brown‐headed Cowbird *Molothrus ater*	1	49	SC2	< 1
Dark‐eyed Junco *Junco hymalis*	1	19	SC1	< 1
Pine Siskin *Spinus pinus*	1	13	SC1	< 1
Brown Creeper *Certhia americana*	1	8	SC1	< 1
Unidentified passerine	7 [5]	NA	NA	3
Unidentified SC1 passerine	26 [6]	25	SC1	12
Unidentified SC2 passerine	3 [1]	65	SC2	1
Violet‐green Swallow *Tachycineta thalassina*	1	21	SC1	< 1
Green‐winged Teal *Anas carolinensis*	1	380	SC3	< 1
California Quail *Callipepla californica*	5	186	SC3	2
Rock Pigeon *Columba livia*	2	345	SC3	1
Northern Flicker *Colaptes auratus*	1	132	SC3	< 1
Eastern Cottontail *Sylvilagus floridanus*	6	900	SC3	3
Eastern Gray Squirrel *Sciurus carolinensis*	2	400	SC3	1
American Red Squirrel *Tamiasciurus hundsonicus*	1	230	SC3	
Unidentified mammal	3	NA	NA	1

^a^ At least 88 (42%) prey items were young‐of‐the‐year, including 33 American Robins, 30 European Starlings, 5 House Sparrows, 4 House Finches, 2 Spotted Towhees, 1 Bewick's Wren, 1 Steller's Jay, 1 American Crow, 5 Unidentified SC1 passerines, 2 Unidentified SC2 passerines, 1 Violet‐green Swallow, 2 California Quail, and 1 Eastern Cottontail Rabbit.

In Wisconsin, we identified individual, uniquely color‐marked breeding male hawks and their delivered prey to species and age (i.e., adult or young‐of‐the‐year) via spotting scope and binoculars. Age of avian prey in our two study areas was determined by color markings and structural attributes of prey (e.g., speckling on upper breast feathers occurs in hatching year but not adult American Robins ( *Turdus migratorius* ); and similar extent of simultaneous partial growth among sheathed flight feathers in the wing [primaries and secondaries] and tail [rectrices] which occurs exclusively in nestling and fledgling birds; see Bielefeldt et al. 1992; Rosenfield et al. 1995; and Cava et al. 2012). Young‐of‐the‐year mammals in both study areas were aged by comparatively smaller size including proportionately small length of tails; we did not age Northern Short‐tailed Shrews ( *Blarina brevicauda* ). Due to the breeding phenology of prey items, there were virtually no young‐of‐the‐year American Robins, Mourning Doves, nor Eastern Chipmunks available as prey during the pre‐incubation stage in Wisconsin (Cutright et al. 2006; RNR, pers. obs.). We highlight that native mammalian diversity is low throughout Vancouver Island, and there are no chipmunks and few other native ground dwelling (diurnal) mammals similar in size to chipmunks as potential prey in urban or rural environments throughout the island (Cowan and Guiguet 1956; Naughton 2012).

Deliveries of post‐fledging prey items in Wisconsin occurred during 22 opportunistic brief visits (~15–40 min), and 12 randomly chosen 2‐ to 3.5‐h visits during 0600–1930 h near an urban nest with five fledged young aged 33 days (3 July) through 67 days (6 August) on the campus of the University of Wisconsin‐Stevens Point, 2022. Age of fledged young was determined at banding by backdating from estimated nestling ages of the oldest chick based on plumage development of known‐age birds (Rosenfield 2018). We climbed to nests during mid‐to‐late June to obtain brood counts and age and band young in both study areas (Rosenfield 2018).

### 2.5 Data Analysis

We report prey items by frequency and their (rounded) proportions of known and/or unidentified species, prey type (i.e., avian or mammalian), and age (young‐of‐the‐year [i.e., nestling or fledgling] or adult). We summarize from Cava et al. (2012) the proportions of adults among the three markedly predominant avian prey species—the American Robin, European Starling ( *Sturnus vulgaris* ), and House Sparrow—during pre‐incubation in Victoria, British Columbia. The total collective number of these three species (*n* = 183) represents 89% of the grand total of 206 ageable urban prey items reported for March and April in Cava et al. (2012).

To facilitate discussion about the size of prey among studies, we report average body mass of identifiable species and categorize prey items into three a priori size classes (SC) using estimated mass of adult and juvenile prey species following our previous research in Wisconsin (Bielefeldt et al. 1992) and British Columbia (Cava et al. 2012; and see below): SC1 ≤ 27 g, SC2 = 28–91 g, and SC3 = ≥ 92 g. We assumed averages of 25 g and 65 g for unidentified birds in SC1 and SC2, respectively (Bielefeldt et al. 1992; Cava et al. 2012); and we estimated 70 g for the American Robin in rural Vancouver Island, British Columbia (Aldrich and James 1991).

Our bases for describing and analyzing diet and foraging ecology of breeding Cooper's Hawks, including the qualitative correlations between body size and aerial agility of bird‐eating raptors and the size and agility of their adult, especially avian prey follow Storer (1966), Reynolds (1972), Wattel (1973), Pennycuick (1975), Snyder and Wiley (1976), Andersson and Norberg (1981), Bielefeldt et al. (1992), and Tornberg et al. (1999, 2014). Concordantly, we use mean body mass of breeding male Cooper's Hawks, and average body masses and size classes (range of body mass) of their prey as ordinal indices to agility. Regarding prey, we qualitatively assume, for example, that adult birds are more agile than fledglings, that smaller adult birds are more agile than larger counterparts, and that adult mammals are less agile than adult birds of similar mass—and more agile than inexperienced young‐of‐the‐year mammals in evading capture by breeding male Cooper's Hawks.

However, these prey agility ordinations may present important limitations in non‐qualitative correlative analyses, such as the potential use of regression‐based modeling to investigate the relationship between body size of predator and prey agility. For example, we have precise, accurate estimates of mean body mass from relatively large, long‐term, systematic samples of captured breeding male Cooper's Hawks near their nests during the nestling stage in our study areas (Rosenfield et al. 2010, 2024), but, like other raptor researchers, we do not have such precise field measurements of delivered avian prey species or their cohorts. Indeed, many of the hawk‐prey studies cited herein, including ours (e.g., Bielefeldt et al. 1992; Millsap et al. 2013; Miller et al. 2022), use estimates of mean biomass from Dunning (2008) as proxies to size of identifiable avian prey species. This source often lacks large, systematic samples of concurrent temporal and/or geographic coverage of prey species and their cohorts (e.g., age) to specific sites, including our study areas; thus, each of these sampling components likely has “measurement error” that is difficult (if not impossible) to assess (Zimova et al. 2023; Rosenfield et al. 2024). Inclusion of this error in model‐based algorithms to regress precise metrics for predator body size on imprecise prey body mass (agility) values in our study is, we believe, at least questionable (*sensu* Hutto 2016).

Further, unlike avian prey in this study, adult and young of the year Eastern Chipmunks and Thirteen‐lined Ground Squirrels typically evade predators by fleeing to narrow, traditionally used underground tunnels (Naughton 2012), and thus in part use ‘complete’ concealment, rather than agility to evade hunting Cooper's Hawks. Notably, the Eastern Chipmunk made up ~30% of the prey deliveries during the pre‐incubation stage (Table 1) and ~34% of prey deliveries during the nestling stage in Wisconsin Cooper's Hawk nests (Bielefeldt et al. 1992); the Thirteen‐lined Ground Squirrel comprised 21% of male deliveries to fledged hawks (see Table 1). Hence, using derived, ordinal metrics other than mean body mass to index prey agility given the anti‐predator life histories in these ‘subterranean hiding’ mammals appears problematic (e.g., what integers would precisely represent the apparent ‘biological’ disparity in ‘agility’ during escape behaviors between these two mammals?). To be clear, we do not believe that agility in breeding male Cooper's Hawks is substantively driven by concealment per se (as described above) acting as a selective pressure via prey escape behavior. Regardless, not accounting conceptually and/or analytically for a differing life history of even a single prey species (or its cohorts) that is disproportionately represented in a multi‐species sample could conceivably render (linear) assumptions about prey agility in a regression model invalid (*sensu* Hutto 2016), and statistical inferences and hence biological conclusions therefrom untenable (e.g., Haskell 1995; Bielefeldt and Rosenfield 1997).

Brood sizes are from successful nests wherein one young was at least ~16–19 days of age (Rosenfield 2018). We regard nests as independent events across years. We describe the distribution of brood sizes in urban and rural nests in Vancouver Island via range, means for region of central tendency, and standard deviation (SD) to index variation around mean values.

We used StatXact‐Turbo (Mehta and Patel 1992) to calculate Chi‐square exact probability in comparing proportions of avian and mammalian prey delivered by uniquely marked males (one item per male) during pre‐incubation in Wisconsin, and a *t*‐test (Wilkinson 1992) to determine if average brood sizes per successful nest differed significantly between urban and rural environments in British Columbia. Significance was assessed at *p* < 0.05.

## 3 Results

### 3.1 Wisconsin

We did not detect prey items other than birds and mammals during the pre‐incubation and post‐fledging stages at 56 and 1 nest(s), respectively (Table 1).

We identified to species 88% of 56 total prey items, one each delivered by a uniquely color‐marked male to a putative mate on 56 separate territories during pre‐incubation, and 97% of 61 prey items delivered by another marked male to post‐fledglings at a different territory (Table 1). There were 10 total known species in the two nesting stage samples. The percent frequency of birds and mammals regardless of species among all prey items, 70% and 30%, and 74% and 26%, were similar between pre‐incubation and post‐fledging periods, respectively (Table 1).

Proportions of birds and mammals during pre‐incubation at urban nests (*n* = 35) and rural nests (*n* = 21) were 74% (*n* = 26) and 26% (*n* = 9), and 62% (*n* = 13) and 38% (*n* = 8), respectively; these proportions did not vary significantly between these two environments (*X* ^2^_1_ = 0.45, *p* = 0.38).

Two ground‐foraging birds, the American Robin, which comprised 32% and 28%, and the Mourning Dove, at 13% and 16%, were the first and third most frequent prey items in the pre‐incubation and post‐fledging stages, respectively (Table 1). Two ground‐foraging small mammals, the Eastern Chipmunk (28%) during pre‐incubation and the Thirteen‐lined Ground Squirrel ( *Spermophilus tridecemlineatus* ) (21%) in the post‐fledging stage, were the second most frequent species among all prey items (Table 1). Collectively, these three species comprised 73% and 67% of all prey items in the pre‐nestling and post‐fledging stages, respectively (Table 1). All pre‐incubation prey items were adults, and at least 48% (*n* = 29; 15 birds and 14 mammals) of all prey items at the post‐fledging stage were young‐of‐year (Table 1).

Proportions of SC1 prey among all prey items during the pre‐incubation and post‐fledgling periods were 17% and 7%, respectively. Proportions of SC2 and SC3 prey items among all prey items were 41% and 68%, and 45% and 25% during pre‐incubation and post‐fledging stages, respectively (Table 1).

### 3.2 British Columbia

We did not detect prey items other than birds and mammals at urban and rural nests in Vancouver Island (Cava et al. 2012; Table 2, this study).

One hundred eighty‐three (89%) of the total 206 total identifiable prey items delivered by male Cooper's Hawks to putative mates in the Victoria study area during pre‐incubation were comprised of three SC2 bird species, the American Robin (*n* = 92), the European Starling (*n* = 63), and House Sparrow (*n* = 28) (these are re‐summarized data from Cava et al. 2012; see METHODS). The proportion of adults in each of these respective species' totals was 90% (83/92), 100% (63/63), and 86% (24/28). Hence, 93% (170/183) of items identifiable to age among these three bird species during pre‐incubation were adults.

Birds comprised 94% of 210 prey items delivered by males during incubation through the post‐fledging stages at 14 rural nests in Vancouver Island, British Columbia (Table 2). We identified species in 81% of all prey items. Three ground‐foraging SC2 birds, the American Robin, European Starling, and House Sparrow, comprised 36%, 17%, and 7% (collectively 60%) of all prey items; unidentified small (SC1) passerines comprised 12% of all prey (Table 2). We identified to species 9 of 12 total mammalian prey items, including Eastern Gray Squirrel ( *Sciurus carolinensis* ), American Red Squirrel ( *Tamiasciurus hudsonicus* ), and Eastern Cottontail ( *Sylvilagus floridanus* ) (Table 2).

Mean number of young per successful nest was 3.64 (SD = 1.07, range 1–5 young, *n* = 400) and 3.69 (SD = 1.09, range = 1–6, *n* = 59) at urban vs. rural nests, respectively. There was no significant difference between these averages (*t* = −0.384, df = 457, *p* = 0.7).

## 4 Discussion

There has been a recent increase in research of habitat use, including predator–prey and niche dynamics in the avivorous, forest‐adapted Cooper's Hawk due to its rapid recovery from the ill effects of various anthropogenic factors during the last several decades of the 1900s. Its high trophic status, ubiquitous presence, high nesting densities, and reproductive success, especially in cities throughout North America, may render it a good model for investigating food webs, including the effects of urbanization on wildlife via comparative studies of urban vs. rural environments (e.g., Kettel et al. 2018; Cooper, Yeh, and Blumstein 2022; Cooper, Schultz, et al. 2022; Miller et al. 2022). In fact, it is conceivable that an abundance of suitable avian prey was propitious for the colonization and heightened reproductive success of urban Cooper's Hawks (Cooper, Schultz, et al. 2022; Morozov 2022).

However, we contend that the above studies are compromised in part due to a lack of fundamental knowledge about this predator's diet and foraging ecology across its range; especially lacking are diet studies of the crucial pre‐incubation stage, wherein territorial males attract potential mates via courtship feeding. These inadequacies are in part revealed through mischaracterizations of its diet and modeling absent the interpopulation variation in body sizes of a raptor that exhibits marked sexual size dimorphism (Rosenfield et al. 2022, 2024). In fact, foraging success of breeding males, who are the principal provisioners of food to their much larger mates and young throughout the nesting cycle, is likely influenced by matching their size with the body size, and hence the agility of their avian prey, though the latter relationship has been challenged (e.g., Bielefeldt et al. 1992; Millsap et al. 2013). Also, few paired studies have documented that urban populations of Cooper's Hawks exhibit higher reproductive output compared to rural populations.

In the following three subsections we discuss, respectively: (1) select comparative and salient aspects of the breeding diet in our study areas and elsewhere, (2) our support for the premise that seasonal agility of prey is plausibly important to (and influences) the size of male breeding Cooper's Hawks, and (3) our failure to support the suggestion that productivity is greater at urban vs. rural nests. We incorporate comments in each section regarding pertinent themes in the technical literature that we hope will clarify concepts, correct mischaracterizations, and expand our knowledge of the diet and foraging ecology of breeding Cooper's Hawks.

### 4.1 Comparative and Salient Aspects of Diet of Breeding Populations of Cooper's Hawks

There were similarities in prey use during pre‐incubation and post‐fledging stages in Wisconsin and British Columbia to prey use in the nestling stage in many parts of the Cooper's Hawk's range, including Maryland, Michigan, New York, Pennsylvania, North Dakota, and our earlier work (1981–1989) in the same Wisconsin study areas herein, as reviewed by Rosenfield (2018), and Rosenfield, Madden, et al. (2020). For example, excluding the ground feeding Mourning Dove (119 g) used by Wisconsin hawks, predominate prey were ground foraging, small‐to‐medium sized, SC2 songbirds weighing between ~25 g and 90 g and, while not so in rural British Columbia, secondarily, ground‐foraging mammals about 100 g (especially rodents) (Table 1). Although researchers have documented breeding Cooper's Hawks preying upon > 80 species, only a few species seem to dominate the diet of nesting Cooper's Hawks within populations during the entire nesting cycle, including our British Columbia and Wisconsin study areas.

Three ground‐foraging species dominated frequency of prey use in each of our two nesting stage samples in Wisconsin: the American Robin and Mourning Dove in pre‐incubation and post‐fledging periods, along with the Eastern Chipmunk and the Thirteen‐lined Ground Squirrel in these respective nesting stages (Table 1). There was no significant variation in proportions of avian and mammalian prey delivered by 56 different males during pre‐incubation between objectively found urban and rural environments throughout the southeastern quarter of Wisconsin, 1991–2023. Similarly, and in previous Wisconsin research (1980–1989), the American Robin, Eastern Chipmunk, and Mourning Dove were the first, second, and third most delivered prey species in the diet of nestling Cooper's Hawks, respectively. Considering all species, SC2 birds and the SC3 (≥ 90 g) Mourning Dove dominated the diet of nestlings, and the overall proportion of birds and mammals in the earlier study was 61% and 39%, respectively (Bielefeldt et al. 1992, 1998).

In British Columbia, three SC2 species, the American Robin, European Starling, and House Sparrow, dominated prey use during pre‐incubation and other nesting stages of urban and rural Cooper's Hawks in British Columbia, respectively (we note the sparrow is about half the weight of the other two species; Cava et al. 2012; Table 2, this study). Compared to Wisconsin where birds comprised about 60%–70% of all prey deliveries across all nesting stages and habitats (Bielefeldt et al. 1992; Table 1, this study), there were much higher proportions of avian prey delivered throughout all nesting stages in urban and rural nests in Vancouver Island, about 96% (Cava et al. 2012) and 94% (Tables 1 and 2), respectively.

Notably, all prey deliveries by males during pre‐incubation in Wisconsin, both avian and mammalian, were adults (Table 1). Similarly, 93% of the three dominant avian prey species delivered by British Columbia males to females during the pre‐laying stage were adults (see Results and Cava et al. 2012).

In summary: males predominately deliver avian prey to nests in both our study populations, but birds are more prevalent in British Columbia (~95%) vs. Wisconsin nests (~60%–70%); adult prey markedly dominates deliveries during the pre‐incubation stage, whereas young‐of‐the‐year cohorts of pre‐laying prey species co‐dominate prey deliveries to nestlings and fledgling hawks in both study areas; comparatively smaller birds are delivered by British Columbia vs. Wisconsin males; and, unlike British Columbia where mammals are rarely delivered, small to medium‐sized mammals, the Thirteen‐lined Ground Squirrel and especially the Eastern Chipmunk are co‐dominant prey throughout the nesting cycle in Wisconsin.

### 4.2 Agility of Prey and Its Possible Seasonal Influence on Body Size of Breeding Male Hawks

Our findings of prey use in overlapping years from widely spaced morphologically different nesting populations of Cooper's Hawks in Wisconsin and British Columbia, and wherein the latter study site breeding males (296 g) are on average ~10% and significantly smaller than Wisconsin counterparts (327 g), support the hypothesis that agility via prey size influences body size of breeding male Cooper's Hawks (Rosenfield et al. 2010; Sonsthagen et al. 2012). Indeed, during the crucial pre‐incubation stage, males on both study areas predominately caught experienced adult birds, but smaller British Columbia males caught proportionately more avian prey, including birds of smaller size and therefore prey of greater aerial agility vs. those taken by larger Wisconsin counterparts. Notably, the collective estimated mean mass of the three predominant prey species in Wisconsin, American Robin, Eastern Chipmunk, and Mourning Dove (~100 g), is about double that for the three dominant British Columbia prey species, American Robin, European Starling, and House Sparrow (~57 g) (Tables 1 and 2).

Hence, breeding male Cooper's Hawks were seasonally obliged to track the escape tactics including greater agility in evasive maneuvering by experienced adults, especially avian prey in both British Columbia and Wisconsin. Such maneuvering is adaptive in male Cooper's Hawks via the likely selective pressure of the agility of small avian prey on this predator's morphology, especially its body size. Further, the concordant difference in male size with prey size between our study populations underscores the principle that avivorous predator species match the size of their adult prey (Snyder and Wiley 1976; Andersson and Norberg 1981; Rosenfield et al. 2010; Sonsthagen et al. 2012). Similarly, “rapid” (< 50 years) decreases in size of male, congeneric Eurasian Goshawks ( *A. gentilis* ) in Finland were “likely” due to the result of selective, including seasonal pressures associated with switching from hunting larger to smaller sized avian prey following decadal changes in prey communities (Tornberg et al. 1999, 2014). We note that we have no evidence of dietary shifts in prey species, nor changes in body size of adult males during our multi‐decadal studies of nesting Cooper's Hawks in British Columbia (1995–2011) and Wisconsin (1980–2023) (Rosenfield and Bielefeldt 1999; Rosenfield et al. 2013, 2024; Rosenfield, Hardin, et al. 2020).

We reiterate that our discovery of large geographic scale, population‐specific variation in body size of breeding male (and female) Cooper's Hawks is essentially unaddressed in modeling exercises regarding habitat suitability and niche investigations (e.g., Cooper, Yeh, and Blumstein 2022; Cooper, Schultz, et al. 2022; Miller et al. 2022; Zuckerberg et al. 2022; Rosenfield et al. 2024). Additionally, and despite available but uncited publications reporting the likely and adaptive tracking of size of avian prey by male *Astur* hawks (e.g., Tornberg et al. 1999, 2014; Rosenfield et al. 2010, 2019), a recent review paper on size dimorphism in raptors indicated that “no empirical data have been provided” that support the hypothesis that body size of male hawks is influenced by the agility of their prey (Schoenjahn et al. 2020). These apparent oversights are concerning given that predator–prey interactions, especially in avivorous raptors, are inherently body size‐dependent such that success of predators depends on their phenotypic match with size of their primary prey and its availability in suitable habitat (Abrams 2000; Rosenfield et al. 2010; Nakazawa et al. 2013; Tornberg et al. 2014). Notably, a single derived metric of body mass (429.67 g) was recently used to index size of all continental male and female Cooper's Hawks in a study of niche dynamics (Miller et al. 2022). To our knowledge this value would likely not represent the weight of any breeding male Cooper's Hawk throughout its range. In fact, population‐specific, average male masses are at least ~100 g less than this derived mark which also would portray the mass of only a few smaller nesting females in some breeding populations (Rosenfield, Madden, et al. 2020; Rosenfield et al. 2024). Ecological modeling absent the biologically important, interpopulation variation in body sizes of breeding males whose foraging success predominately drives reproductive output may compromise our ability to understand how prey selection may be linked to variation in habitat quality throughout the vast breeding range of Cooper's Hawks.

**FIGURE 2:**
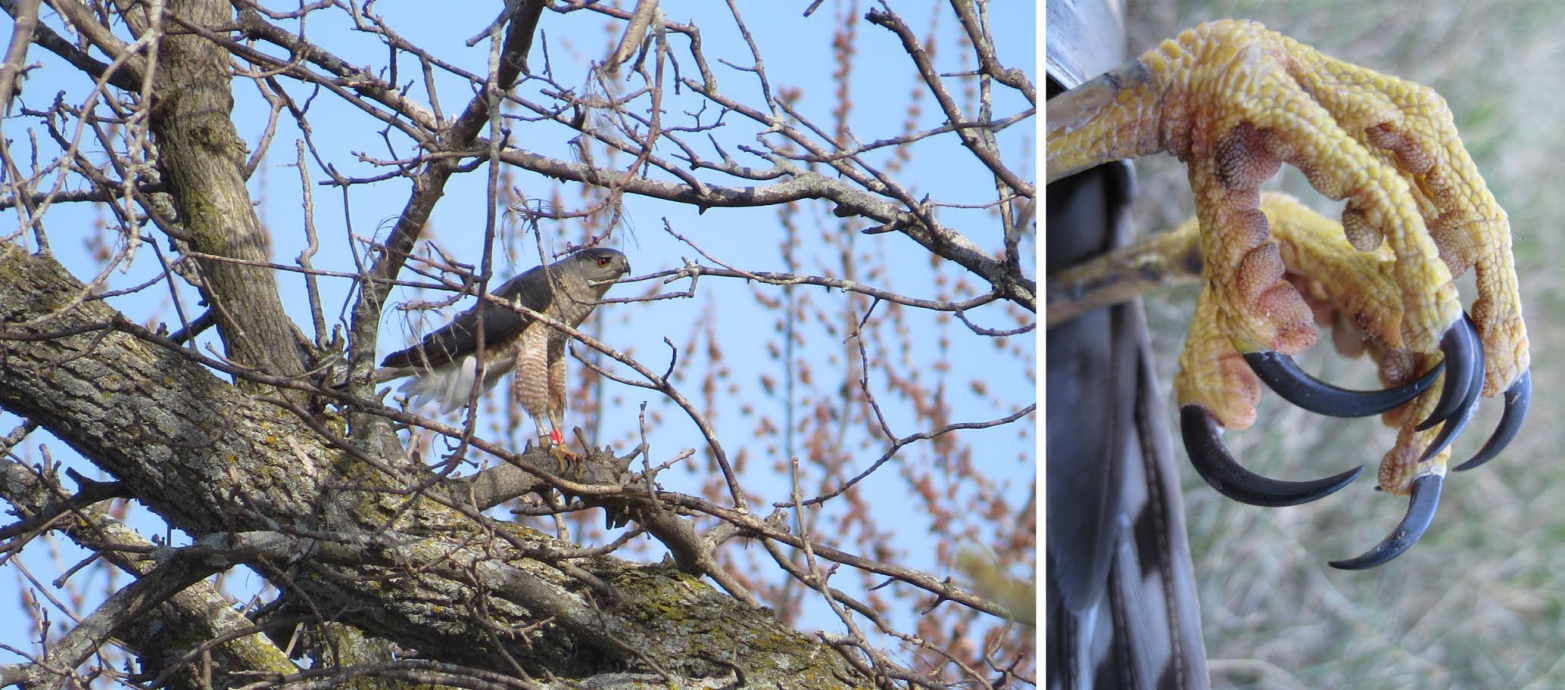
Note the long legs and toes that enhance reach of agile bird prey in the Cooper's Hawk.

Accentuating the principle of body size‐ or agility‐adaptive predator–prey dynamics is that the American Robin, *the* predominant prey of nesting Cooper's Hawks in our British Columbia and Wisconsin samples, exhibits a relatively smaller continental body size along coastal British Columbia (Aldrich and James 1991; Vanderhoff et al. 2020) where some of the smallest North American breeding Cooper's Hawks occur (Rosenfield, Madden, et al. 2020). Further, we recently reported that relative to body size, male Cooper's Hawks nesting in British Columbia have, and contrary to the findings of Whaley and White (1994), relatively long toes compared to that ratio in larger Wisconsin counterparts (Rosenfield, Stewart, et al. 2020). Long toes in accipiters enhance reach for agile tender‐skinned avian prey in mid‐air (Wattel 1973; Figure 2), which morphology would be adaptive in capture success in smaller British Columbia males whose diet is comprised of smaller and more agile avian prey. Less agile mammalian prey are more prevalent in the diet of Wisconsin birds, which may suggest less selective pressure for relatively long toes in that population (Rosenfield, Stewart, et al. 2020). Additionally, it is conceivable that compared to females, the markedly smaller size of male Cooper's Hawks in part “pre‐adapted” (Cooper, Schultz, et al. 2022) them to foraging abundant urban songbirds, which may have influenced the rapid colonization of cities by breeding Cooper's Hawks in only about two or three decades since about 1970 (Stout and Rosenfield 2010; Rosenfield, Madden, et al. 2020).

That male Cooper's Hawks exhibit adaptive, aerial agility concordant with the evasive escape flight tactics of seasonally available adult songbirds is further exemplified via the similar extent (~7 days) of advancement across 36 years (1980–2016) in this hawk's timing of egg laying with the American Robin's earlier arrival time in spring migration in Wisconsin (plucked feathers of American Robins are the most frequently found prey remains during our multi‐decadal pre‐incubation searches for breeding Cooper's Hawks [Rosenfield et al. 2013, 2016; Rosenfield, Hardin, et al. 2020]). The temporal synchrony and dominant frequency of robins as pre‐incubation prey was consistent with the state's warmer springs due to global warming (Rosenfield et al. 2016). By contrast, in many birds the lack of a correlation between rates of warming in breeding and wintering areas likely caused phenological mismatches between the presence of breeders and the appearance of their prey on breeding grounds, which resulted in reduced reproductive output and/or population declines, including some raptors (Jones and Creswell 2010; Halupka et al. 2023). However, Wisconsin Cooper's Hawks responded favorably to the effects of global warming via increased productivity, likely due to their advanced phenology congruent with earlier migration of adult avian prey (Halupka et al. 2023). (Notably the American Robin, a habitat generalist, and the most frequent prey item in the pre‐incubation stage, was the fifth most abundant bird species [among 229] throughout Wisconsin, including the most frequently reported species among statewide habitats, rural and urban, in North American Breeding Bird Survey routes, 1995–2000 [Cutright et al. 2006]). The above factors likely allowed Cooper's Hawks to acquire resources during pre‐incubation to lay larger‐sized clutches in *both* urban and rural environments in Wisconsin (this study; Rosenfield et al. 2016; Halupka et al. 2023). Similarly, Snyder and Wiley (1976) indicated that a sudden increase in migrant, medium‐sized (~30–50 g) adult songbirds “triggered” egg‐laying by Cooper's Hawks in rural Arizona due to the abundance of food for egg production.

### 4.3 Possible Influence of Avivory on Reproductive Output

We reiterate that ground squirrels and chipmunks, especially the Eastern Chipmunk, are common prey in the diet of nestling Cooper's Hawks throughout much of their range, including Wisconsin (Bielefeldt et al. 1992; Rosenfield, Madden, et al. 2020). That said, Millsap et al. (2013) indicated that “a key factor” limiting productivity of Cooper's Hawks in rural north Florida (2.8 fledged young per successful nest) vs. elsewhere among North America habitats (range = 2.7–4.0 young per successful nest as summarized in Rosenfield, Madden, et al. 2020) was the lack of a small ground squirrel, or other small ground‐foraging mammals (in the community and hence) in this raptor's diet. Moreover, Millsap and colleagues stated that “some” urban Cooper's Hawk populations maintain high nest production without using mammalian prey, but that this was “likely possible because of the greater abundance of suitable avian prey species in urban environments.” Assumed high abundance levels (or concentrations) of avian prey in cities have been pitched to explain relatively high productivity at nests of avivorous, urban‐breeding raptors, including Cooper's Hawks in Victoria, British Columbia, and several Wisconsin cites (e.g., Rutz 2008; Rullman and Marzluff 2014; Kettel et al. 2019; Morozov 2022).

Interestingly, native mammalian diversity is low throughout Vancouver Island, and there are no chipmunks and few other native ground dwelling (diurnal) mammals similar in size to chipmunks as potential prey in urban or rural environments throughout the island (Cowan and Guiguet 1956; Naughton 2012). Yet the average brood size per successful rural Cooper's Hawk nest on Vancouver Island (3.7) where predominate prey is birds (Table 2) is among the highest for the species; and virtually identical and non‐significant to the average brood size (3.6) found in this isle's city of Victoria during the same study years, 1995–2011. To be clear, the lack of ground‐dwelling mammals in the diet of nesting rural Cooper's Hawks in Vancouver Island did not appear to limit their productivity. Thus, we are intrigued as to why average brood sizes of rural, north Florida Cooper's Hawks (~2.8 fledged young) were much lower than those in rural British Columbia (3.7 young) wherein both rural study areas there is a high reliance on avian prey. (We note that [Millsap et al. 2013] reported the number of fledged young per nest, which counts are likely not directly comparable to our report of number of advanced‐aged nestlings per nest [see METHODS]. Counts of fledged young may be biased low due to reduced detection probabilities of fledged Cooper's Hawks [Rosenfield and Sobolik 2014]).

Notably, in our multi‐decadal research of Cooper's Hawks in Wisconsin, we have found no evidence that habitat variation, including urban vs. rural, was related to variation in body mass of breeding adults, indices of reproductive success, nesting phenology, nesting density, production of recruits, nestling sex ratios, adult survivorship, or adult fidelity to breeding territories (Rosenfield 2018; Rosenfield et al. 2016; Rosenfield, Madden, et al. 2020; Rosenfield, Hardin, et al. 2020). Similarly, there was no significant difference in the proportions of avian and mammalian prey in urban vs. rural nests during pre‐incubation in Wisconsin. Our demographic and prey data from both British Columbia and Wisconsin do not support conjecture that an assumed higher urban avian prey base generates higher productivity in urban vs. rural nests of Cooper's Hawk (Millsap et al. 2013; Kettel et al. 2018; Morozov 2022).

Researchers in north Florida suggested that “the lack of a small ground squirrel component in the prey community was a key factor limiting the reproductive potential” of breeding Cooper's Hawks (Millsap et al. 2013). This investigation was novel in reporting that breeding males exhibited a “heavy reliance” on procuring food via hunting nestling birds in nest boxes and canopy tree nests. However, a conceivable alternative explanation regarding a link between lower prey availability and lower brood sizes was reduced numbers of avian, not mammalian prey. Indeed, in central Florida and during the same study years as Millsap's investigation, Barve et al. (2024) reported that snake activity near the ground quadruples during March through June, which in turn increases snake predation at ground‐dwelling songbird nests during this raptor's pre‐incubation and nestling stages. High rates of snake predation could conceivably reduce ground‐dwelling songbird numbers and hence limit resources for egg production and/or reduce hatchling survival via starvation (starvation is a major source of mortality in raptors; Newton 1979). Unfortunately, these north Florida findings, which were reported to be broadly applicable across much of the southeastern United States into eastern Texas (Millsap et al. 2013), did not include clutch sizes (i.e., reproductive potential) or hatchling survival rates.

Variation in population‐specific life‐history factors other than prey type and availability can influence reproductive output of Cooper's Hawks. For example, we reported comparatively lower average brood counts (3.0 young) in 80 rural Cooper's Hawk nests in the prairie and croplands of west‐central North Dakota (vs. ~3.7 in British Columbia and Wisconsin in concurrent study years; Rosenfield et al. 2007). And although sample sizes of prey at only two nests in this North Dakota landscape were relatively small, mammals, including the Thirteen‐lined Ground Squirrel, made up 30% (70% birds [predominately passerines 25–40 g]) of the diet of nestling Cooper's Hawks (Peterson and Murphy 1992). We indicated that the lower average brood size in this rural population was not due to higher hatchling mortality rates compared to those in other northern populations. Rather, lower brood sizes in North Dakota were linked to comparatively lower average clutch sizes (3.5 eggs/clutch vs. 4.3 and 4.4 in Wisconsin and British Columbia, respectively), due possibly to a trade‐off between resources routed to endogenous reserves depleted in individuals returning after spring migration vs. reserves used for egg production. Notably, the North Dakota population begins incubation about 2 to 3 weeks later than Wisconsin and British Columbia Cooper's Hawks at about the same latitude (Rosenfield et al. 2007). Moreover, we also suggested that food availability was likely not limiting in North Dakota because rural breeding populations were increasing and nesting densities were comparatively high and similar to those in Wisconsin, British Columbia, and, interestingly, Millsap's north Florida study area (Rosenfield et al. 2007, 2019; Taylor et al. 2021).

We reiterate that there are few detailed studies of the diet of nesting Cooper's Hawks among the varied habitats of their vast North American range (Rosenfield, Madden, et al. 2020; Rosenfield et al. 2022). Thus, it is particularly concerning when researchers cast range‐wide, untenable generalizations about this species' diet and foraging behavior. For example, it has been suggested that there is “strong evidence” that urban Cooper's Hawks “specialize exclusively on adult life‐stages of larger‐bodied birds” (Malone et al. 2017), and that compared to rural counterparts, urban Cooper's Hawks “switch to a more specialized diet of larger prey birds” (Zuckerberg et al. 2022). Yet the small (28 g) House Sparrow, including adults and young of the year, is reported as common or relatively frequent prey of nesting Cooper's Hawks in several cities and rural areas throughout North America (Cava et al. 2012; Rosenfield et al. 2022).

Similarly, we agree with Morozov (2022) who indicated that urban ecology has paid “insufficient attention” to the existence of synergistic effects of ecological factors, including the major roles of predation pressure and temporal feeding conditions to the successful colonization of cities by Cooper's Hawks vs. the influence of these and other life history factors on the reproductive demographics in rural populations. Especially lacking are paired urban vs. rural studies, and unfortunately rural breeding populations appear to be less recently studied than urban Cooper's Hawk populations (Rosenfield, Madden, et al. 2020; Rosenfield et al. 2022).

Regardless, we have shown that researchers and land managers should not assume a consistent relation between the extent of reproductive output at nests in urban Cooper's Hawk populations based solely on the presumed or known proportions of avian and/or mammalian prey in their diet (this study; Rosenfield 2018; Rosenfield, Madden, et al. 2020). Indeed, in a recent study researchers indicated that “once established in urban landscapes” Cooper's Hawks apparently produce larger clutches due to the influence of “high concentrations” of “preferred” avian prey (Zuckerberg et al. 2022). Yet to our knowledge, there are no tenable natural history findings underscoring such a general relationship. We know of no clutch size data to support this claim. More precisely, and excluding Wisconsin research (Rosenfield 2018; Rosenfield, Madden, et al. 2020), we are unaware of any Cooper's Hawk study in which investigators systematically counted clutch sizes in urban and rural nests during the incubation period. We reiterate that analyses of demographic data including egg and brood counts from 40 years statewide in Wisconsin do not (and contra to incorrect reporting of 1.1–1.2 “more chicks” in this state's urban vs. rural nests in a review paper [Kettel et al. 2018]) show that variation in reproductive output of Cooper's Hawks is linked to habitat variation, including urban vs. rural environments (e.g., Rosenfield and Bielefeldt 1999; Rosenfield et al. 1995, 2000, 2016; Rosenfield, Hardin, et al. 2020). The above and other inaccurate and/or misleading statements about the diet and foraging ecology of Cooper's Hawks can compromise analyses of habitat use that in part assume links between body size and other structural attributes of hawks with availability and/or use of suitable prey (e.g., Cooper, Schultz, et al. 2022; Miller et al. 2022).

Lastly, we highlight that efficient wildlife conservation depends increasingly on a good understanding of the processes by which animal populations colonize or persist in human landscapes (McKinney 2002), especially those environments in constant flux and influenced by novel stressors due to increasing anthropogenic factors, including urbanization and climate change (Cooper, Yeh, and Blumstein 2022; Cooper, Schultz, et al. 2022; Halupka et al. 2023).

## Author Contributions

**Robert N. Rosenfield:** conceptualization (lead), data curation (lead), formal analysis (lead), funding acquisition (lead), investigation (lead), methodology (lead), project administration (lead), resources (lead), software (lead), supervision (lead), validation (lead), visualization (lead), writing – original draft (lead), writing – review and editing (lead). **Andrew C. Stewart:** conceptualization (lead), data curation (lead), formal analysis (lead), funding acquisition (lead), investigation (lead), methodology (lead), project administration (lead), resources (lead), software (lead), supervision (lead), validation (lead), visualization (lead), writing – original draft (lead), writing – review and editing (lead). **Paul N. Frater:** conceptualization (supporting), data curation (lead), formal analysis (lead), funding acquisition (supporting), investigation (supporting), methodology (equal), project administration (supporting), resources (supporting), software (lead), supervision (equal), validation (lead), visualization (supporting), writing – original draft (supporting), writing – review and editing (lead). **Eric L. Holmgren:** conceptualization (supporting), data curation (supporting), formal analysis (supporting), funding acquisition (supporting), investigation (supporting), methodology (supporting), project administration (supporting), resources (lead), software (supporting), supervision (supporting), validation (lead), visualization (supporting), writing – original draft (supporting), writing – review and editing (supporting).

## Conflicts of Interest

The authors declare no conflicts of interest.

## Data Availability

Data on prey items in British Columbia and Wisconsin, and brood sizes at urban and rural nests in British Columbia are available at Zenodo: https://zenodo.org/records/16747349.
